# Distally Unlocked Intramedullary Nailing With Cement Fixation for Impending and Actual Pathologic Humerus Fractures: A Retrospective Case Series

**DOI:** 10.5435/JAAOSGlobal-D-20-00090

**Published:** 2020-06-12

**Authors:** Richard A. Pizzo, Tyler Hoskins, Jay N. Patel, Justin M. Miller, David Goyette, Christopher Mazzei, James C. Wittig

**Affiliations:** From the Division of Orthopaedic Trauma & Complex Adult Reconstruction, Department of Orthopaedic Surgery, Jersey City Medical Center, Jersey City, NJ (Dr. Pizzo and Dr. Miller), and the Division of Orthopaedic Oncology & Sarcoma Surgery, Department of Orthopaedic Surgery, Morristown Medical Center, Morristown, NJ (Mr. Hoskins, Dr. Patel, Mr. Goyette, Mr. Mazzei, and Dr. Wittig).

## Abstract

The humerus is a common site of metastatic tumor involvement and pathologic fracture. Intramedullary nailing is a treatment option that offers the benefit of protecting a long segment of diseased bone, but it is not without complications. This study aims to examine the survival, functional outcomes, and complications of patients treated with cement-augmented unlocked intramedullary nailing for actual and impending pathologic fractures of the humeral shaft.

**Methods:**

From 2014 to 2019, 26 patients were treated with this technique. Functional outcomes were assessed using the Musculoskeletal Tumor Society scoring system. Outcome scores, complications, reoperations, and mortality were determined by retrospective chart reviews and direct patient examinations.

**Results:**

The mean age at the time of surgery was 66.8 years. The mean follow-up was 20.2 months. Patients reported significant improvement in the mean Musculoskeletal Tumor Society score from 10.5 preoperatively to 26.1 after surgery (*P* < 0.001). Five patients died of disease during the follow-up period. One patient had intraoperative fracture propagation during implant placement, and one patient experienced a postoperative rotator cuff tear.

**Discussion:**

Unlocked intramedullary nailing with cement augmentation is a reliable treatment method for actual and impending pathologic fractures of the humerus that results in favorable outcomes, including consistent pain relief and restoration of function.

Bones are the most common site of metastasis in patients with cancer, and the humerus is a frequently involved site.^1,2^ Primary lesions may also arise in the humerus, although they are less common. Metastatic tumors are destructive to local bony architecture, causing microfractures that generate a notable amount of pain and compromise the biomechanical integrity of the bone, increasing the risk of pathologic fracture.^1^ The humerus is the second most common site of fractures in long bones due to metastatic bone disease, accounting for about 20% of all pathologic fractures.^3^

Pathologic fractures pose a substantial burden, both to individual patients, and on an economic level to the healthcare system. Patients who sustain pathologic fractures have much higher morbidity, and costs of treatment of pathologic fracture are notable. The projected cost to the US healthcare system in treating metastatic skeletal disease is 12 billion dollars annually.^4^ Despite this expenditure, patients with skeletal metastasis generally have poor survival and the presence of skeletal-related events, such as pathologic fractures, portends a worse prognosis.^5,6^ The reported survival time after pathologic humerus fracture is between 3 and 10 months, although newer treatment therapies have increased the survival rates.^7^ Because these lesions typically occur late in the disease process when patients exhibit notable systemic disease burden, the goals of treatment are rapid restoration of a painless and functional extremity, rather than curative.

Several surgical options exist for treating pathologic fractures of the humerus, including plate and screw osteosynthesis, endoprostheses, and intramedullary nails. Many studies exist exploring these treatment options, but little consensus exists on the optimal fixation method.^8,9,10,11,12^ Open reduction and internal fixation with plate and screw constructs, although providing effective fracture stabilization, does not protect the entire bone and may actually increase fracture risk in adjacent bones because of the stress-riser effect at the plate edge.^13,14^ The clinical significance of stabilizing a long segment of bone is difficult to quantify, however, and studies comparing open reduction and internal fixation and intramedullary nailing have demonstrated similar outcomes.^15^ One advantage of intramedullary nailing is that it allows for preservation of the patients native bone and muscle attachments. This is in contrast to reconstruction with a megaprosthesis, which is a much more invasive procedure, and has been associated with more complications, greater blood loss, and longer hospital stays.^9,16^ When intramedullary fixation is used, distal interlocking screws can potentially be a source of complications because this region of the extremity contains a number of major neurovascular structures in closeness, near the necessary instrumentations that are subject to damage during the procedure.^17,18,19,20,21^ These complications can be circumvented by using cement fixation for distal rotational control instead of distal interlocking screws. The aim of this study was to assess the clinical and functional outcomes of patients treated with distally cemented intramedullary nails for pathologic fractures or impending fractures of the proximal humerus and humeral shaft.

## Methods

A retrospective review was conducted on all patients presenting to one of two academic medical centers from 2014 to 2019 with actual or impending pathologic fractures of the humerus. Patients who had pathologic fractures, disease involving the cortices leading to cortical compromise of >50%, or pain due to the lesions were indicated for surgery. All patients treated with distally unlocked, cemented intramedullary nailing with a minimum of 6-month follow-up were included in this study. Patients with lesions within the medullary canal of the humeral or lesions <2 cm without cortical compromise were treated conservatively and were excluded from the study. Patients with follow-up of less than 6 months were also excluded from the study. Ethical review board approval was obtained before study initiation. Patients enrolled in the institutional study registry were screened for inclusion. Functional outcomes were assessed using the Musculoskeletal Tumor Society (MSTS) upper extremity scoring system.^22^ Outcomes for each patient were evaluated at two weeks, six weeks, three months, six months, and then on an as-needed basis. Radiographs were obtained at each of these time points to assess for signs of local recurrence, peri-implant fracture, or hardware failure. Mortality was tracked through medical records. Outcome scores, complications, and reoperations were determined by retrospective chart review, direct patient examination, review of longitudinally maintained institutional database. Statistical analysis was performed using the Wilcoxon signed-rank test.

**Figure 1:**
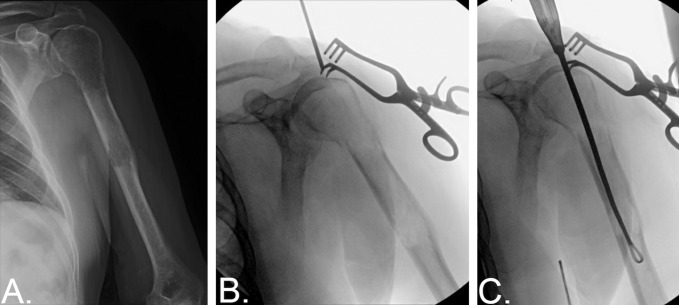
**A**, Radiograph of the humerus demonstrating lytic lesions of both the humeral shaft and proximal humerus with notable cortical compromise, resulting in nondisplaced pathologic fracture of the surgical neck. **B**, Intraoperative fluoroscopy demonstrating identification of the starting point for instrumenting the humerus. **C**, Intraoperative fluoroscopy depicting the use of a looped curet through the starting point in the humeral head to grossly debulk the tumor.

**Figure 2:**
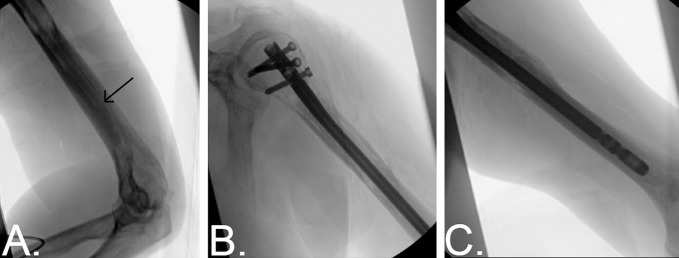
**A**, Intraoperative fluoroscopy showing cement being injected into the distal humeral shaft. Arrow indicates the tip of the cement gun nozzle. B/C. Intraoperative fluoroscopy demonstrating the proximal (**B**) and distal (**C**) aspects of the final cemented intramedullary nailing construct.

All surgical procedures were performed under general anesthesia. The patient was positioned supine with a bump positioned under the ipsilateral scapula. An incision was made overlying the anterolateral aspect of the acromion and carried through the skin and subcutaneous tissue. A deltoid splitting approach was used, and the starting point for the intramedullary implant was identified under fluoroscopy. A small rent was made in the rotator cuff tendon in line with its fibers once the starting point was determined, as shown in Figure 1, B. Next, the opening reamer was used to gain access to the medullary canal. If the pathologic lesion was accessible through this incision, rongeurs and curettes were used to remove/scrape gross neoplasm vigorously from the endosteal surface (Figure 1, C). If the lesion occurred more distally, had a soft-tissue component, or was in the setting of an actual displaced fracture, then an accessory incision was made over the area of the tumor or fracture site to allow for curettage of the neoplasm. Surrounding medullary bone was also débrided of tumor, both proximally and distally to the fracture site, when possible. A biopsy specimen for each patient was sent for frozen section. A guidewire was next placed down the humeral shaft into the distal humeral fragment and confirmed on fluoroscopy before reaming. A long cement gun nozzle was placed over a guidewire down the intramedullary canal into the distal humerus. The guidewire was then removed. Cement was then vigorously mixed and poured into the cement gun. The cement gun was then connected to the nozzle already within the intramedullary canal. The cement was injected under fluoroscopy while it was in a puddy-like state, as shown in Figure 2, A. The distal third of the intramedullary canal was filled with cement. In patients with impending fractures, cement was injected up to the level of the lesion. In patients with actual fractures, the fracture site was spared of cement, and the cement was confined to the distal 1/3 of the canal. The cement gun nozzle was slowly backed out because the cement was injected down the canal. The humeral intramedullary nail was then inserted under fluoroscopy in an antegrade manner. Two to three proximal interlocking screws were then placed through the implant, Figures 2, B and C. The rent in the rotator cuff was repaired with Ethibond suture. The wounds were copiously irrigated and closed.

All patients were instructed to remain non–weight-bearing to the affected upper extremity postoperatively and placed in a sling for comfort. At 2 weeks after surgery, the sling was discontinued, and patients were instructed to begin pendulum exercises and perform gentle range of motion of the shoulder and elbow. Patients were allowed unrestricted range of motion exercises at 4 weeks. At 6 weeks, patients were allowed progressive weight-bearing and to begin strengthening exercises.

## Results

### Demographics

Twenty-six patients were identified for potential inclusion. Nine of these patients were excluded for not meeting the minimum follow-up criteria of greater than 6 months. Seventeen patients were included in the final analysis: nine patients with impending pathological fractures and eight patients with actual fractures. Demographic information is shown in Table 1. Of these patients, five died because of their advancing neoplastic disease. Nine men and eight women were included in this cohort. The mean age at the time of index procedure was 66.8 years (range, 19 to 86). The mean follow-up was 20.2 months (range, 6 to 52.8). Five patients had primary skeletal lesions, and 12 had metastatic disease at the time of presentation. Diagnoses included the following: renal cell carcinoma (2), thyroid adenocarcinoma, pharyngeal carcinoma, myeloma (5), lymphoma, breast adenocarcinoma (3), lung adenocarcinoma (2), prostate adenocarcinoma, and vulvar squamous cell carcinoma.

**Table 1:** Selected Patient Demographics

Age, y, mean (SD)	66.8 (12.1)
Sex, n (%)	
Male	9 (52.9)
Female	8 (47.1)
Fracture status, n (%)	
Fracture	8 (47.1)
Impending	9 (52.9)
Laterality, n (%)	
Left	6 (35.3)
Right	11 (64.7)
Lesion type, n (%)	
Primary	5 (29.4)
Metastatic	12 (70.6)
Pathology, n (%)	
Myeloma	5 (29.3)
Breast adenocarcinoma	3 (17.6)
Lung adenocarcinoma	2 (11.8)
Renal cell carcinoma	2 (11.8)
Lymphoma	1 (5.9)
Thyroid adenocarcinoma	1 (5.9)
Prostate adenocarcinoma	1 (5.9)
Pharyngeal carcinoma	1 (5.9)
Vulvar squamous cell carcinoma	1 (5.9)

### Outcomes

**Figure 3:**
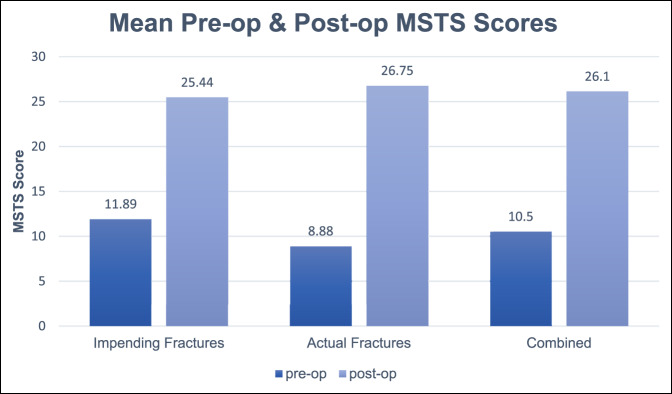
The mean MSTS score of patients with pathologic fractures and impending fractures before surgery versus at final follow-up. MSTS = Musculoskeletal Tumor Society

The mean improvement in the MSTS score before and after surgical treatment was 15.6 points, *P* < 0.001. The mean preoperative MSTS score of all patients was 10.5 (range, 7 to 21). The mean postoperative MSTS score for all patients at the most recent follow-up visit was 26.1 (range, 19 to 29), *P* < 0.001. Patients with pathologic fractures had, on average, a greater improvement in the MSTS score than patients with impending fractures (17.86 versus 13.56, Figure 3). No notable difference was observed in postoperative MSTS scores for patients with impending versus actual pathologic fractures (25.44 versus 26.75, respectively). One patient had an intraoperative fracture propagation while advancing the intramedullary nail. Another patient suffered a rotator cuff tear approximately 4 months after surgery while carrying a heavy object. His pain and motion improved with physical therapy. No cement-related pulmonary complications, postoperative fractures or failures, neurovascular injuries, wound complications, or infections in any patient were observed. Five patients died from their disease throughout the study period (time frame to death, 6 to 17 months). Before surgery, 14 patients received some form of the following neoadjuvant therapy: chemotherapy (11), radiation (0), and chemotherapy + radiation (3). Postoperatively, 15 patients received the following adjuvant treatment: chemotherapy (6), radiation (2), and chemotherapy + radiation (7). Radiation treatment to the humerus was administered at an average of 4 weeks after surgery to help eradicate any residual neoplastic disease.

## Discussion

Pathologic fractures are relatively common and a notable source of morbidity in patients with skeletal metastases. The humerus is the second most commonly affected long bone after the femur.^2^ In addition, because advances in oncologic treatment continue to increase life expectancy in patients with malignancies, an associated increased risk of skeletal metastasis and pathologic fractures was observed, and knowing how to treat these challenging injuries will become increasingly important. This series demonstrates that distally cemented intramedullary nailing seems to be a safe and effective treatment method for pathologic fracture and impending fracture of the humerus. The goals of treatment of these lesions are rapid restoration of a functional, painless extremity. This is particularly important because these patients tend to have a large systemic disease burden and may experience increased dependence on upper extremities because of tumor involvement in other limbs. Stable fixation allowing immediate use and weight-bearing as tolerated, while subsequently minimizing complications, is of paramount importance.^5-7^ The patients in this series undergoing distally cemented intramedullary nailing had a mean improvement in the MSTS score of 15.6 points and a mean postoperative MSTS score of 26.1 (range, 19 to 29), signifying these goals were effectively achieved, *P* < 0.001. Both patients with impending and actual fractures reported a notable improvement in pain postoperatively and increased range of motion and overall function of the extremity. This technique offers several advantages over other treatment options, including protecting a long segment of diseased bone from future insult, avoiding risks to local neurovascular structures by distal locking screws, and augmenting fixation with cement.

Another major advantage of the construct used in this series is that it avoids the potential complication of neurovascular insult associated with distal interlocking screws. Complications may arise using the “perfect circle” technique to place distal locking screws in a small area of the body riddled with major nerves and vessels, namely the radial nerve, median nerve, and lateral antebrachial cutaneous nerve. Numerous cadaveric studies demonstrate notable anatomic proximity to these structures by placing screws in such a fashion. Screw trajectories have been reported to come within millimeters and/or directly traverse through these nerves.^18,19^ In a case series of 51 humeral fractures treated with intramedullary nailing and distal locking screws by Blyth et al,^20^ 8 patients (15.7%) suffered an iatrogenic nerve palsy to either the radial or lateral antebrachial cutaneous nerves. Furthermore, Baltov et al^21^ reported a 2.7% iatrogenic nerve palsy to the same nerves in a similar series of 111 patients treated with distal interlocking screws. By relying on cement augmentation, the need for distal locking screws for rotational control is obviated, and these neurovascular complications can be avoided.

Eliminating the need for distal locking screws also reduces surgical time and fluoroscopy time. In a series of 122 patients with humeral fractures treated via intramedullary nailing with or without distal locking screws, Colombi et al^23^ found that the surgical time was notably shorter on average (63 versus 88 minutes) when leaving the nail unlocked distally. In addition, the average fluoroscopy time was notably shorter (59 versus 100 seconds) as well. These benefits of shorter surgical time and fluoroscopy time did not correlate with a difference in outcomes; the study found no difference in union, pain, or shoulder, and elbow scores between the cohorts. Limiting surgical time in these patients is important because they invariably carry notable medical risk because of the nature of their metastatic disease.

The nonunion rate for pathologic fractures remains extraordinarily high, up to 65%, because the biologic environment is altered.^24-26^ This is a notable source of pain and limitation in function. Because many of these fractures will not proceed to union, augmenting with polymethylmethacrylate bone cement is a strategy to increase the rigidity and stability of the construct, eliminating the need for bony healing for mobility and pain control. In a retrospective series of 21 patients with pathologic humerus fractures, Laitinen et al^10^ found that patients with cement-augmented intramedullary nails had less pain and better functional restoration immediately after surgery compared with their noncemented counterparts. Using polymethylmethacrylate cement in this manner has been shown to improve construct rigidity and stability, allowing for immediate weight-bearing, decreasing subsequent fracture risk, reducing tumor burden through thermal damage, and slowing tumor progression.^27,28^ All of these factors likely contribute to the notable improvement in pain and rapid return of function observed in this series. Cement augmentation has historically been postulated to be associated with increased risk of wound complications because it necessitates opening of the fracture site, although no such complications were observed in this cohort.^10^

This study has several limitations. Two of the most notable limitations of this analysis are the relatively small sample size and a mean follow-up of less than 2 years. This is partly because of the nature of the pathology studied in this series. These patients have bony metastases, which signifies stage 4 cancer and has a poor prognosis in many cases, limiting overall survivability and thus mean follow-up. Additional limitations of this series are the retrospective nature and lack of a control group. Future randomized controlled trials are needed to further elucidate the benefits of this technique.
